# Combined hyperbaric oxygen therapy and repetitive transcranial magnetic stimulation in depression and PTSD: future perspectives

**DOI:** 10.4103/mgr.MEDGASRES-D-25-00089

**Published:** 2026-01-06

**Authors:** Jakub Tlapák, Eliška Tlapáková, Adam Pásler, Dittmar Chmelař, Michal Hájek

**Affiliations:** The Institute of Aviation Medicine, Prague, Czech Republic; Faculty of Biomedical Engineering, Czech Technical University in Prague, Czech Republic; Department of Psychiatry, Pardubice Region Hospital, Pardubice, Czech Republic; Department of Emergency Medicine and Military General Medicine, Military Faculty of Medicine, University of Defence, Hradec Králové, Czech Republic; Centre of Hyperbaric Medicine, Ostrava City Hospital, Ostrava, Czech Republic; Institute of Laboratory Medicine, Institute of Microbiology, Faculty of Medicine, University of Ostrava, Ostrava, Czech Republic; Centre for Hyperbaric Medicine of Faculty of Medicine University of Ostrava and Ostrava City Hospital, Ostrava, Czech Republic

Major depressive disorder (MDD) and posttraumatic stress disorder (PTSD) are prevalent psychiatric conditions with significant impact on individuals and society. MDD is one of the most common mental disorders affecting more than 250 million people globally. PTSD is the most serious and incapacitating mental disease that can result from trauma exposure with a prevalence greater than 23%.^1^,^2^ It is assumed that the etiopathogenesis of MDD or PTSD is the result of a complex interaction between biological and psychosocial factors. Despite advances in pharmacological and psychotherapeutic interventions, many patients experience inadequate response or side effects, necessitating the exploration of alternative treatments. Therefore, it is necessary to search for other potential therapeutic approaches.

Hyperbaric oxygen therapy (HBOT) involves breathing 100% oxygen in a pressurized chamber exceeding 1 atmosphere absolute (ATA). Both mechanisms enhance tissue oxygenation by increasing arterial oxygen pressure by 9–16 times and extending the diffusion distance of oxygen from the capillaries to the tissues by 3–4 times.^3^ HBOT has been studied for its potential therapeutic benefits in various medical conditions, including neuropsychiatric disorders. Repetitive transcranial magnetic stimulation (rTMS) is also a non-invasive neuromodulation technique that has garnered significant attention for its efficacy in treating mood disorders, particularly MDD and PTSD. By applying magnetic pulses to specific brain areas, the mechanism of action is multifaceted and differs between MDD and PTSD, involving complex neurobiological processes.^4^ While the precise mechanisms are still being explored, current evidence supports rTMS and HBOT as promising isolated therapeutic approaches for alleviating symptoms in patients with these disorders. Further research is crucial to better understand the underlying neurobiological mechanisms and to optimize treatment protocols for specific patient populations.

However, the purpose of this article is to summarize the mechanisms through which HBOT and rTMS, as a combination therapy, can alleviate the symptoms of MDD and PTSD. Furthermore, to emphasize the safety of these two therapeutic methods. However, it is important to recognize that HBOT and rTMS are not standalone solutions and should be considered as part of a comprehensive treatment plan, including psychotherapy and pharmacotherapy.

**Mechanism of action of repetitive transcranial magnetic stimulation in major depressive disorder and posttraumatic stress disorder:** In MDD, dysregulation of brain network connectivity, particularly in the prefrontal cortex (PFC), contributes significantly to persistent negative mood and cognitive impairments. The PFC, responsible for higher-order executive functions and emotional regulation, often exhibits reduced activity in individuals with depression. rTMS targets this region by inducing electric currents in cortical neurons, leading to alterations in neuronal firing patterns and enhanced synaptic plasticity. High-frequency rTMS (≥ 5 Hz) applied to the left dorsolateral prefrontal cortex increases cortical excitability, facilitating improved communication between the PFC and subcortical regions, such as the amygdala and hippocampus, which are involved in emotion regulation and memory. This restoration of cortical-subcortical interactions is believed to alleviate depressive symptoms by enhancing cognitive flexibility and emotional regulation. On a molecular level, rTMS has been shown to affect the release of neurotransmitters, such as serotonin, dopamine, and norepinephrine, which are often disrupted in MDD.^4^,^5^ Additionally, rTMS stimulates the production of essential proteins for neuronal survival, growth, and neuroplasticity.

In PTSD, the response of the brain to traumatic memories is often disrupted, with increased activity in the amygdala and decreased activity in the PFC, leading to impaired regulation of emotional responses to trauma-related stimuli. Similar to MDD, rTMS is used to modulate brain activity; however, in PTSD, it is typically applied to the dorsolateral prefrontal cortex and the right PFC. High-frequency rTMS targeting the left dorsolateral prefrontal cortex aims to enhance inhibitory control over the hyperactive amygdala, thereby reducing emotional reactivity to traumatic memories. Low-frequency rTMS (≤ 1 Hz), often directed at the right PFC, helps reduce excessive cortical activity, which may contribute to heightened emotional responses in PTSD patients.^4^,^6^ Neuroimaging studies have shown that rTMS can help restore the balance between these regions, improving emotional regulation and alleviating symptoms such as hyperarousal, intrusive thoughts, and avoidance behaviors. Additionally, rTMS may stimulate neuroplasticity, facilitating the rewiring of trauma-related neural circuits in the brain and promoting long-term improvements in emotional processing and resilience.

**Mechanism of action of hyperbaric oxygen therapy in major depressive disorder and posttraumatic stress disorder:** HBOT exerts its therapeutic effects in MDD and PTSD through several mechanisms, starting with enhanced oxygenation and neuroplasticity. This enhanced oxygenation stimulates the release of growth factors essential for synaptic plasticity and neuronal survival. The ability of the brain to form new neural connections, which is often impaired in MDD and PTSD, is facilitated by this process, helping to restore functional connectivity and cognitive abilities. Another critical mechanism by which HBOT affects psychiatric disorders is the reduction of oxidative stress. Both MDD and PTSD are associated with an imbalance between reactive oxygen species and antioxidants, contributing to neuronal damage and inflammation. HBOT has antioxidant properties, reducing reactive oxygen species production and enhancing the activity of antioxidant enzymes.^3^,^7^ This mitigates oxidative stress, protecting the brain from further injury and supporting recovery. By reducing oxidative damage, HBOT helps to preserve brain structure and function, which is vital for improving the symptoms of these disorders.

HBOT also exerts significant anti-inflammatory effects, addressing the chronic inflammation observed in MDD and PTSD. Elevated pro-inflammatory cytokines have been linked to mood dysregulation and heightened stress responses. HBOT has been shown to decrease the levels of these cytokines while increasing the production of anti-inflammatory mediators. This reduction in inflammation may alleviate the neuroinflammatory processes contributing to the onset and persistence of psychiatric symptoms, further aiding in the restoration of normal brain function and emotional regulation. Additionally, HBOT promotes neurogenesis, particularly in the hippocampus, a region crucial for memory and emotional regulation, which is often impaired in these conditions. This effect enhances hippocampal function, contributing to improved cognitive and emotional outcomes in patients with MDD and PTSD.^3^,^7^

**Current evidence on the effect of hyperbaric oxygen therapy and repetitive transcranial magnetic stimulation:** HBOT increases the expression of neurotrophic factors, particularly brain-derived neurotrophic factor, which supports the growth, differentiation, and survival of neurons.^3^,^8^ rTMS, by modulating cortical excitability, stimulates brain-derived neurotrophic factor-dependent synaptic plasticity and alters network activity in the brain.^4^,^5^ HBOT reduces neuroinflammation by decreasing the levels of pro-inflammatory cytokines (tumor necrosis factor-α, interleukin-1β, and interleukin-6) while increasing anti-inflammatory cytokines (interleukin-10). It also reduces oxidative stress by increasing superoxide dismutase and catalase.^3^,^8^,^9^ rTMS reduces the neuroinflammatory activity of microglia and astrocytes and influences cytokine levels, thereby reducing both systemic and central inflammation.^5^ HBOT improves brain energy metabolism and modulates glutamatergic, dopaminergic, and GABAergic signaling.^8^,^9^ rTMS alters the activity of neuronal circuits, including increasing dopaminergic and glutamatergic activity in the PFC during high-frequency stimulation.^4^,^5^ HBOT, at increased partial oxygen pressure, enhances oxygen delivery to ischemic and hypoperfused areas of the brain, promoting neuronal regeneration.^3^,^8^ rTMS increases regional cerebral blood flow, particularly in the PFC, which correlates with therapeutic effects in the treatment of MDD.^5^

**Figure 1:**
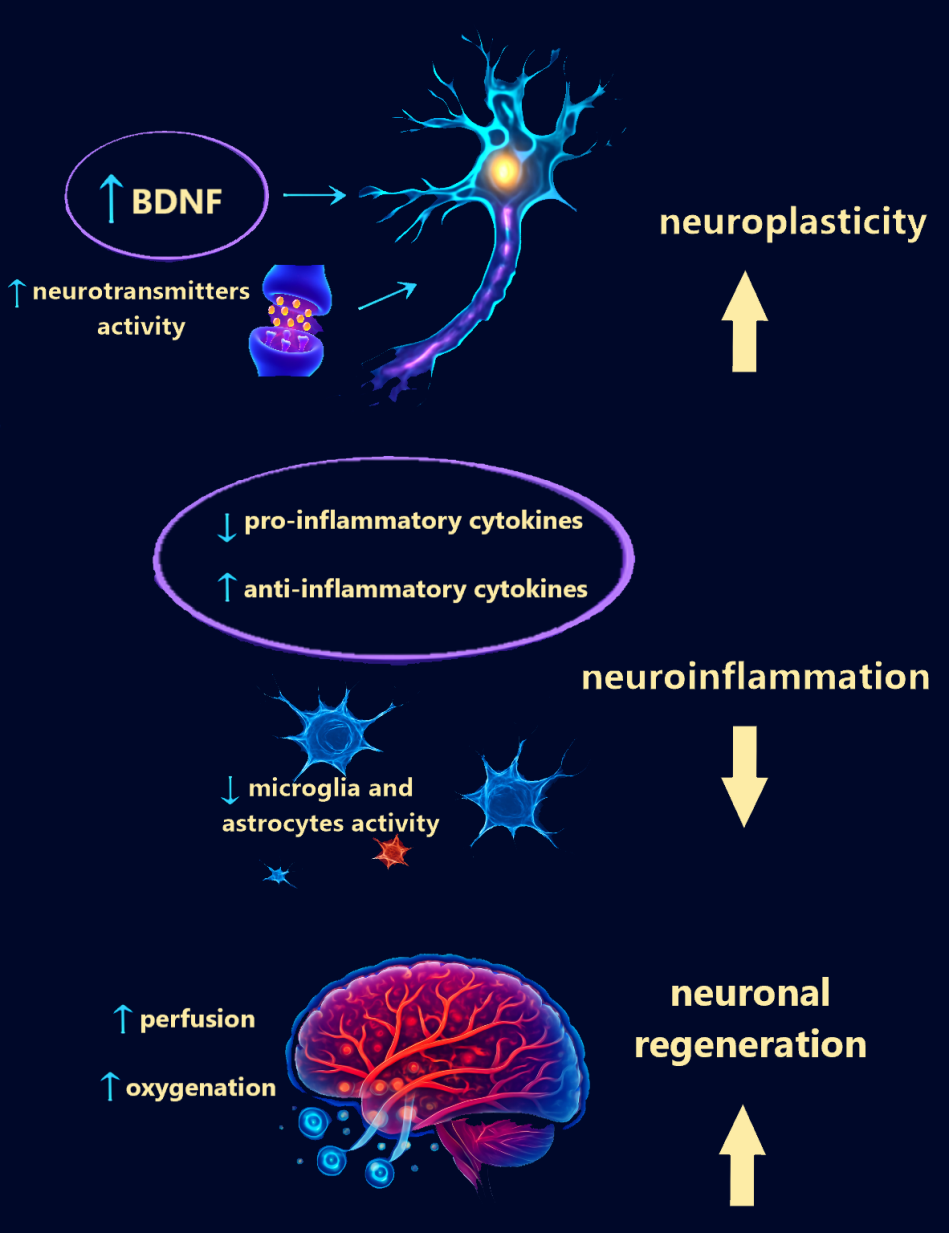
Summary of the main overlapping or synergistic effects of the mechanism of potential use of combined hyperbaric oxygen therapy and repetitive transcranial magnetic stimulation therapy in major depressive disorder and posttraumatic stress disorder. Modified by Tlapák J based on OpenAI ChatGPT, 2025. BDNF: Brain-derived neurotrophic factor.

Both methods promote neurogenesis and the reorganization of neuronal connections, have anti-inflammatory and neuroprotective effects, influence dysregulated neurotransmitter systems, and improve brain perfusion and oxygenation. The overlapping or synergistic effects of the mechanism are illustrated in **Figure 1**.

A search of scientific databases including Web of Science, PubMed, and Scopus did not yield any studies investigating the use of combined therapy with HBOT and rTMS in either MDD or PTSD. According to the search results, this combination therapy has so far only been applied in studies addressing the treatment of motor dysfunctions or cognitive deficits of traumatic or non-traumatic origin, chronic pain syndromes, as well as tinnitus and auditory function impairments.

Zhang et al.^10^ applied a combination of HBOT and 5 Hz high-frequency rTMS in 94 patients with post-stroke dysphagia, achieving a significant success rate of 89.36%. The article, published in 2025, concludes that this combined therapy effectively improves post-stroke dysphagia by stimulating the cortical area corresponding to the suprahyoid muscle group.^10^

Wang et al.^11^ published data from a randomized controlled trial (RCT) investigating the clinical efficacy and mechanism of action of high-frequency rTMS combined with HBOT in the treatment of chronic migraine. In the study, 52 patients treated with combination therapy showed significant reductions in headache severity, frequency of headache attacks, duration of single attacks, and the number of analgesics taken at the end of the treatment course and one month after treatment, compared to the control group. The study concluded that high-frequency rTMS combined with HBOT demonstrates a strong clinical effect in treating chronic migraine and provides a preventive effect against recurrence, with a lasting therapeutic benefit. The mechanism of action primarily involves the regulation of pain-related factors, such as serotonin, calcitonin gene-related peptide, and β-endorphin, in plasma or cerebrospinal fluid.^11^

Wang et al.^12^ evaluated the clinical effects of HBOT combined with rTMS in patients with motor dysfunction following spinal cord injury. In this RCT, 49 patients were treated with combined therapy. After treatment, the American Spinal Injury Association Impairment Scale motor score, ASIA sensory index score, and activities of daily living scores in both groups increased, with the scores in the combined therapy group being significantly higher than those in the control group. The study concluded that HBOT combined with rTMS promotes the recovery of motor and sensory functions and improves the ability to perform activities of daily living in spinal cord injury patients with motor dysfunction.^12^

Fei et al.^13^ evaluated in a RCT the efficacy of HBOT combined with rTMS on patients with cognitive impairment after cerebral infarction. 36 patients were assigned to the combined therapy group with static improvement in results of mini-mental state examination, Montreal cognitive assessment, and Barthel index. National Institute of Health stroke scale was significantly lower. Blood flow and flow velocity of the combined group were higher. Data were evaluated between groups, before and after treatment. The conclusion of this study is that HBOT in combination with rTSM can effectively improve the cognitive function and hemodynamic indexes of patients with cognitive impairment after cerebral infarction, thus improving the efficacy of rehabilitation.^13^

Xie et al.^14^ in their single-center, assessor-blind, RCT for HBOT and rTMS combined therapy protocol in vascular cognitive impairment with four parallel arms (control group, HBOT group, rTMS group, and HBOT combined with rTMS group) published in 2023, in the future, perhaps more will be revealed about the potential benefits. The results of the study have not yet been published.^14^

**Adverse effects of repetitive transcranial magnetic stimulation and hyperbaric oxygen therapy:** Despite the relative safety of rTMS, certain adverse effects have been reported that may limit its use or necessitate individualized adjustments to the treatment protocol. The most common side effects include transient headaches (occurring in up to 28–39% of patients) and discomfort at the coil application site, typically attributed to stimulation of superficial scalp muscles. These symptoms are generally mild to moderate in intensity and resolve spontaneously within a few hours. Other frequently observed reactions include dizziness, mild nausea, fatigue, and temporary cognitive changes, most notably short-term impairment of concentration, tinnitus, or transient alterations in auditory perception in the absence of adequate protection against coil-generated noise.^15^

The most serious potential risk associated with rTMS is the induction of a seizure, although its incidence is extremely low. This risk increases in individuals with a history of epilepsy, brain injury, or those taking medications that lower the seizure threshold. Other potentially serious reactions may include significant mood alterations (e.g., hypomania in patients with bipolar disorder), autonomic symptoms such as palpitations or transient hypertension, and possible negative effects on neuroplasticity in cases of improperly set stimulation parameters.^15^

Current evidence suggests that rTMS is safe for long-term use when properly administered, although large-scale studies evaluating its effects on brain structure and function over many years are still lacking. The procedure is contraindicated in patients with metallic implants in the cranial region (excluding dental fillings), as well as in individuals with active epilepsy or severe cardiovascular disorders.^15^

Although HBOT is generally considered safe, it may lead to toxic effects, particularly affecting the lungs and the central nervous system. Pulmonary toxicity is typically associated with prolonged exposure to elevated partial pressures of oxygen. The underlying mechanism involves oxidative damage to the alveolar epithelium and capillary endothelium, resulting in inflammation and impaired gas diffusion. The most common manifestations of pulmonary oxygen toxicity include airway irritation, coughing, reduced vital lung capacity, and acute respiratory distress syndrome-like picture in cases of prolonged exposure to high oxygen concentrations.^16^

Oxygen toxicity affecting the central nervous system develops more acutely than pulmonary toxicity and is related to the generation of reactive oxygen species, leading to lipid peroxidation and neuronal excitotoxicity. These effects typically occur at partial pressures exceeding 2 ATA. Clinical manifestations include perioral fasciculations, clonic muscle jerks, visual disturbances such as tunnel vision, and generalized seizures—known as the Paul Bert effect—with an incidence estimated between 0.01% and 0.03%. According to systematic reviews, the overall risk of neurologic adverse events is low; however, it increases in the presence of predisposing factors such as hypercapnia, hypoglycemia, fever, individual sensitivity to hyperoxia, and the use of certain medications or hormones.^16^

Absolute contraindications to HBOT include untreated pneumothorax, acute severe bronchospasm, specific medications, and non-ventilated air-containing spaces such as pulmonary cysts. A careful evaluation is also required in patients with other severe pulmonary conditions, malignancies, or implanted medical devices, including certain types of pacemakers.^16^

No statistically significant adverse effects of the combined therapy of HBOT and rTMS were identified in the aforementioned published studies. However, based on these data, it cannot be ruled out that none exist. Therefore, it is not yet possible to assess which combined therapy protocol (sequential intervention) could be applied to minimize adverse effects. Both HBOT and rTMS are considered safe therapeutic modalities; however, their potential adverse effects necessitate strict adherence to safety protocols and individualized assessment of exposure risk for each patient.

**Conclusion:** HBOT and rTMS share overlapping mechanisms of action. This suggests that their combination could have synergistic effects in the treatment of MDD and PTSD. Furthermore, both are non-invasive and safe therapeutic approaches. However, data on the incidence of adverse effects associated with their combination therapy remain insufficient. Additional studies are needed to achieve a higher level of evidence-based medicine, but this combination therapy appears to be promising for future clinical practice.
